# Air pollution and respiratory diseases: ecological time series: Poluição do ar e doenças respiratórias: estudo ecológico de série temporal

**DOI:** 10.1590/1516-3180.2015.0237250216

**Published:** 2016-07-18

**Authors:** Luiz Fernando Costa Nascimento, Luciana Cristina Pompeo Ferreira Vieira, Kátia Cristina Cota Mantovani, Demerval Soares Moreira

**Affiliations:** I PhD. Researcher, Department of Energy, Universidade Estadual Paulista (UNESP), Guaratinguetá, and Assistant Professor, Department of Medicine, Universidade de Taubaté (UNITAU), Taubaté, SP, Brazil.; II BSc. Postgraduate student, Department of Energy, Universidade Estadual Paulista (UNESP), Guaratinguetá, SP, Brazil.; III MSc. Postgraduate student, Department of Energy, Universidade Estadual Paulista (UNESP), Guaratinguetá, SP, Brazil.; IV PhD. Researcher, Department of Physics, Faculty of Science, Universidade Estadual Paulista (Unesp), Bauru, SP, Brazil.

**Keywords:** Particulate matter, Air pollution, Pneumonia, Bronchiolitis, Mathematical models, Material particulado, Poluição do ar, Pneumonia, Bronquiolite, Modelos matemáticos

## Abstract

**CONTEXT AND OBJECTIVE:**

Exposure to air pollutants is one of the factors responsible for hospitalizations due to respiratory diseases. The objective here was to estimate the effect of exposure to particulate matter (such as PM_2.5_) on hospitalizations due to certain respiratory diseases among residents in Volta Redonda (RJ).

**DESIGN AND SETTING:**

Ecological time series study using data from Volta Redonda (RJ).

**METHODS:**

Data on hospital admissions among residents of Volta Redonda (RJ), between January 1, 2012, and December 31, 2012, due to pneumonia, acute bronchitis, bronchiolitis and asthma, were analyzed. Daily data on PM_2.5_ concentrations were estimated through the CCATT-BRAMS model. The generalized additive Poisson regression model was used, taking the daily number of hospitalizations to be the dependent variable and the PM_2.5_ concentration to be the independent variable, with adjustment for temperature, relative humidity, seasonality and day of the week, and using lags of zero to seven days. Excess hospitalization and its cost were calculated in accordance with increases in PM_2.5_ concentration of 5 µg/m^3^.

**RESULTS:**

There were 752 hospitalizations in 2012; the average concentration of PM_2.5_ was 17.2 µg/m^3^; the effects of exposure were significant at lag 2 (RR = 1.017), lag 5 (RR = 1.022) and lag 7 (RR = 1,020). A decrease in PM_2.5_ concentration of 5 µg/m^3^ could reduce admissions by up to 76 cases, with a decrease in spending of R$ 84,000 a year.

**CONCLUSION:**

The findings from this study provide support for implementing public health policies in this municipality, which is an important steelmaking center.

## RESUMO

**CONTEXTO E OBJETIVO:**

A exposição aos poluentes do ar é um dos fatores responsáveis por internações por doenças respiratórias; o objetivo foi estimar o efeito da exposição a material particulado (como PM_2,5_) sobre as hospitalizações devido a certas doenças respiratórias em moradores de Volta Redonda (RJ).

**TIPO DE ESTUDO E LOCAL:**

Estudo ecológico de séries temporais utilizando dados de Volta Redonda (RJ).

**MÉTODOS:**

Foram analisados dados de internação hospitalar, entre 1 de janeiro de 2012 e 31 de dezembro de 2012, devida a pneumonia, bronquite aguda, bronquiolite e asma, em residentes em Volta Redonda (RJ), e dados diários das concentrações de PM_2,5_ obtidos a partir do modelo CCATT-BRAMS. Utilizou-se o modelo aditivo generalizado de regressão de Poisson, sendo o número diário de internações a variável dependente e a concentração PM_2,5_ a variável independente, ajustado para temperatura, umidade relativa, sazonalidade e dia da semana e defasagens entre zero e sete dias. Excesso de internação hospitalar e seu custo, de acordo com o aumento de 5 ug/m^3^ na concentração de PM_2,5_, foram calculados.

**RESULTADOS:**

Ocorreram 752 internações em 2012; a concentração média de PM_2,5_ foi de 17,2 ug/m^3^; os efeitos da exposição foram significativos em lag 2 (RR = 1,017), lag 5 (RR = 1,022) e lag 7 (RR = 1,020). A redução em 5 ug/m^3^ na concentração de PM_2,5_ poderia reduzir até 76 casos as internações com diminuição nos gastos de R$ 84 mil/ano.

**CONCLUSÃO:**

Os achados deste estudo fornecem subsídios para implantar políticas públicas de saúde neste município, que é importante polo siderúrgico.

## INTRODUCTION

Hospitalizations due to respiratory diseases may result from acute exposure to air pollutants, among other causes. These pollutants are generated by natural sources or anthropogenic sources, and these sources are classified as stationary sources such as power plants and industries, and mobile sources, represented mainly by the vehicle fleet.

In 2012, 1.3 million hospitalizations due to respiratory diseases (chapter 10 of the International Classification of Diseases, ICD, 10^th^ revision) occurred in Brazil. These gave rise to expenditure of approximately R$ 1.2 billion; of these, 64,000 hospitalizations were in the state of Rio de Janeiro, with an expenditure of R$ 55 million (US$ 1 ≈ R$ 2.00 at that time).^1^

Several factors, such as low birth weight, parental smoking, lack of breastfeeding, in addition to the effects of exposure to air pollutants are known to be associated with pneumonia.^2^^,^^3^ Studies in Brazil, both in large cities and in medium-sized cities, have shown that respiratory diseases other than pneumonia are noticeably influenced by the effects of exposure to air pollutants, such as particulate matter less than 10 microns in aerodynamic diameter (PM_10_), nitrogen dioxide (NO_2_), sulfur dioxide (SO_2_), ozone (O_3_) and carbon monoxide (CO), thereby leading to hospitalizations.^4^^,^^5^^,^^6^^,^^7^^,^^8^

These pollutants are usually quantified through monitoring by state environmental control agencies. However, not all places have this monitoring available. Data estimation through mathematical modeling as such as the Chemical Coupled Aerosol and Tracer Transport model for Brazilian developments on the Regional Atmospheric Modeling System (CCATT-BRAMS) can minimize this problem.^9^^,^^10^

CCATT-BRAMS is a mathematical model that makes it possible to perform numerical simulations of weather and climate, by solving for large phenomena explicitly on spatial scales and by parameterizing the processes that occur at scales smaller than the spatial resolution of the model. The Center for Weather Forecasting and Climate Research of the National Institute of Space Research (CPTEC-INPE) does this modeling process on a daily basis, producing daily diagnoses and predictions for up to three days, covering all of South America. It takes into consideration transportation of various gases and aerosol particles, which is estimated from the number and locations of outbreaks of fires that are observed through remote sensors, thus generating daily estimates of various pollutants. The horizontal resolution of this operation is 25 km by 25 km, with 38 atmospheric levels, of which the first level is from ground level to 40 meters above the ground, and this method has already been validate.^10^ Fine particulate matter (PM_2.5_), which accounts for 60 to 70% of PM_10_,^11^ along with carbon monoxide (CO), nitrogen oxides, ozone and volatile organic compounds (VOCs), are the pollutants for which concentrations are estimated through the model. These records are estimated every three hours, daily.

The application of data estimated through this model can be seen in studies developed in Brazil that have correlated exposure to PM_2.5_ with hospitalizations due to pneumonia, other respiratory diseases and cardiovascular diseases.^5^^,^^12^^,^^13^^,^^14^ The importance of studying how exposure to this pollutant can act on human health comes from the fact that, because of its aerodynamic diameter (less than 2.5 microns), it can remain suspended in the air for a longer time and thus be transported over longer distances and also reach deeper portions of the respiratory system.^15^

## OBJECTIVE

The aim of this study was to estimate the effects of exposure to PM_2.5_ in hospitalizations due to pneumonia, acute bronchitis, bronchiolitis and asthma in Volta Redonda (RJ), using data estimated through CCATT-BRAMS.

## METHOD

This was an ecological time series study with data on hospitalizations due to pneumonia (ICD 10^th^ revision, codes J 12.0 - J 18.9), acute bronchitis and bronchiolitis (ICD 10^th^ revision, codes J 20.0 and J 21.9) and asthma (ICD 10^th^ revision, codes J 45.0 J 45.9), among subjects of both sexes at all ages, living in Volta Redonda (RJ). The study period was between January 1, 2012, and December 31, 2012.

### Place of study

Volta Redonda is located at 22° 29 ‘ S and 44° 06 ‘ W, in the Paraíba valley, in the state of Rio de Janeiro, Brazil. The total area of the municipality is 182.8 km^2^, of which 54 km^2^ comprises the main urban area of the municipality. The urban area is located along the banks of the river Paraíba do Sul, and is at an altitude of 350 meters above sea level. Its total population is approximately 260,000 inhabitants with a per-capita income of R$ 27,577.00. It has a vehicle fleet of about 110,000.^16^ Volta Redonda has a mesothermal climate and high relative humidity (77%), even in the colder months, when it ranges from 71% to 72%. The mean annual temperature is 21 °C, with a mean minimum of 16.5 °C and mean maximum of 27.8 °C. The economy of Volta Redonda, while still anchored in industry, also focuses on services and trade. The city houses the National Steel Company (Companhia Siderúrgica Nacional, CSN) and other industries such as factories producing cement, oxygen and nitrogen, flat steel and tin products and is an important link between two highways, which have intense traffic of heavy vehicles and buses. The city has nine hospitals. Three of the hospitals are affiliated to the Brazilian National Health System (Sistema Único de Saúde, SUS) and six are private hospitals. All of these hospitals care for pediatric patients.^16^

The data on hospital admissions due to respiratory diseases, according to place of residence, were taken from the database of the Brazilian Ministry of Health, (DATASUS), through authorizations for hospitalization (AIH) within SUS for the study period, day by day. Data on air pollutants, temperature and relative humidity were estimated using the CCATT-BRAMS model. The pollutant analyzed in this study was the fine particulate matter (PM_2.5_), in µg/m^3^.

Pearson’s correlation test was used to estimate possible correlations between the concentrations of the pollutants and hospitalizations. The effects of exposure to environmental pollutants may be reflected in admissions on the same day or some days later. Therefore, its effects on hospitalizations were investigated on the same day as the exposure (lag 0) and also on the seven subsequent days (lag 1 up to lag 7), because there is no consensus regarding the size of this window. A generalized additive model (GAM) of Poisson regression was used, because the outcome was a discrete quantitative variable. The model was adjusted for seasonality, using the number of days that had elapsed since the start of the study, and for days of the week by means of indicator variables for the days of the week, because there may have been a decrease in hospital admissions on weekends. Minimum temperature and average relative humidity were included in the model. The statistical software used cubic smoothing spline functions for temperature and relative air humidity in order to account for the non-linearity of meteorological variables.

The exponent of the coefficient value provided by Poisson regression was used to calculate the relative risks (RR) of PM_2.5_ exposure and hospital admission due to some form of respiratory disease.

The average, minimum and maximum values and their standard deviations for the number of hospitalizations, PM_2.5_ concentration, temperature and relative humidity were calculated, along with the Pearson correlation values. The relative risk of hospitalization was calculated with 95% confidence intervals. Increases in the relative risk through increases of 5 µg/m^3^ in PM_2.5_ concentration were calculated as percentage values (pp)_._

The expression IRR = exp (β *Δ*pol*) provides the increase in relative risk (IRR), where β is the coefficient given by the Poisson regression and Δ*pol* is the 5 µg/m^3^ increase in the concentration of the air pollutant PM_2.5_.

The population attributable fraction (PAF) was used to estimate the excess number of hospitalizations according to the increase in PM_2.5_, using the expression

PAF = (1- 1/RR) * N

RR is the relative risk and N is the total number of hospital admissions in this expression. The financial costs of possible excess hospital admission was calculated using the PAF value multiplied by the average financial cost of each hospital admission due to respiratory disease, which was also obtained through DATASUS.

The Statistica software was used for the analysis and the significance level used in the analysis was alpha = 5%.

## RESULTS

There were 1560 admissions due to all respiratory diseases in Volta Redonda (RJ), over the study period, and these cost R$ 1.4 million. Hospital admissions with diagnoses of pneumonia, bronchitis, bronchiolitis and asthma accounted for 752 cases, with costs of around R$ 850,000. The average, minimum and maximum values of the variables, and their respective standard deviations, are shown in Table 1.

**Table 1.**
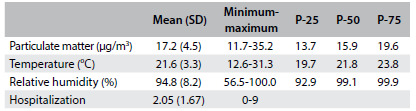
Minimums, maximums, means, standard deviations (SD) and quartiles of the study variables. Volta Redonda (RJ), 2012

P-25 = percentile 25; P-50 = percentile 50; P-75 = percentile 75.

The average PM_2.5_ concentration was 17.2 µg/m^3^, with a minimum of 11.7 and maximum of 35.2 µg/m^3^. On 22 days, the PM_2.5_ concentration exceeded the values considered acceptable by the World Health Organization (WHO). The Pearson correlation coefficient matrix values are shown in Table 2. There was no correlation between the number of hospitalizations and the PM_2.5_ concentrations, but these concentrations were significantly correlated with the temperature and relative humidity values.

**Table 2.**
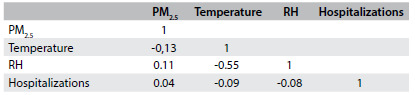
Matrix of Pearson correlation coefficients. Volta Redonda (RJ), 2012

RH = relative humidity.

**Figure 1.**
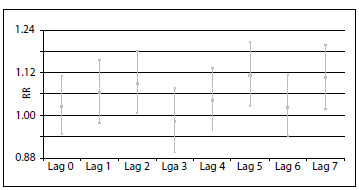
Increase in relative risk according to increment of 5 μg/m^3^ in the concentrations of fine particulate matter. Volta Redonda, 2012.

The values of the coefficients and their standard errors, obtained through Poisson regression using lags of 0 to 7 days, are shown in Table 3. The effects of exposure to PM_2.5_ were seen to be significant two, five and seven days prior to hospitalization, with the following values: lag 2 (RR = 1.017; 95% CI = 1.001-1.034); lag 5 (RR = 1.022; 95% CI = 1.005-1.038); and lag 7 (RR = 1.020; 95% CI = 1.004-1.037). The percentage increases in the relative risks and their 95% confidence intervals, corresponding to lags of zero to seven days for an increase of 5 ug/m^3^ are shown in Figure 1.

**Table 3.**
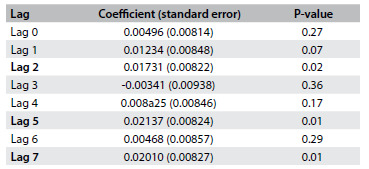
Values of the coefficients and standard errors obtained through the generalized additive model of Poisson regression according to lags from zero to seven days. Volta Redonda (RJ), 2012

This increase in PM_2.5_ concentration was found to result in a significant increase of up to 9 percentage points in the risk of hospitalization due to pneumonia, acute bronchitis, bronchiolitis and asthma. The relative risks according this increase were RR = 1.09 for lag 2 and RR = 1.11 for lag 5 and lag 7, and all of these were statistically significant (P-value < 0.05).

A decrease in PM_2.5_ concentration of 5 µg/m^3^ would, according to the calculation of the population attributable fraction, entail a decrease of 76 hospitalizations. This would lead to savings of around R$ 84,000 per annum, given that the average cost of hospitalization due to these diseases was R$ 1100.00. It should be noted that this reduction in costs related only to hospitalization due to pneumonia, acute bronchitis, bronchiolitis and asthma.

## DISCUSSION

This study, even though it only comprised a one-year time series, showed the relevance of exposure to fine particulate matter, i.e. PM_2.5_, in hospitalizations due to pneumonia, acute bronchitis, bronchiolitis and asthma among people living in Volta Redonda (RJ), which is an important national steelmaking center. Furthermore, it showed that a reduction in the concentration of this pollutant could lead to decreased numbers of hospitalizations and costs to the health system.

Most Brazilian studies have investigated exposure to PM_10_ among hospitalizations due to respiratory diseases, especially pneumonia and asthma.^4^^,^^5^^,^^6^^,^^7^^,^^8^ Unlike in other Brazilian studies, we used data estimated by means of a mathematical model developed by national researchers. We emphasize that this model has been used in other studies developed in Brazil,^5^^,^^12^ but even so, few Brazilian studies have been conducted on PM_2.5_ because there are few instrument stations for monitoring this pollutant.

Fine particulate matter (PM_2.5_) originates from the combustion process of diesel and gasoline-powered vehicles, burning of biomass and burning of coal to generate power. Its composition may vary depending on the place of study and whether its surface composition includes nitrates, sulfates, chlorides, metals such as Na, Al, P, S, Ca or Fe, among others, or polycyclic aromatic hydrocarbons. This composition may vary depending on the place of study.^17^ This pollutant is generated mainly by stationary sources in the case of Volta Redonda, but the vehicular fleet also provides a contribution, given that this city has intense traffic of heavy vehicles crossing the city, because it is an important link between two major highways.

The findings from our study show the deleterious effect of exposure to PM_2.5_ through hospitalizations due to pneumonia, acute bronchitis, bronchiolitis and asthma, in all age groups. The effect was statistically significant on the second, fifth and seventh days after exposure, with relative risks of between 1.017 and 1.022. The relative risk increased to 1.113 (95% CI: 1.026-1.206) with an elevation of 5 µg/m^3^ in the concentration of this pollutant.

In a study using similar methodology, with data estimated through CCATT-BRAMS, in which the study population consisted of children and the average PM_2.5_ concentrations were of the order of 28.6 µg/m^3^, the relative risk was 1.009 (95% CI: 1.001-1.017).^5^ Another study used data from CCATT-BRAMS but included all diseases in chapter 10 of the ICD 10^th^ revision (codes J 00-J 99) and included children under the age of 5 years and elderly people aged over 65 years who were admitted to hospitals affiliated to the Brazilian National Health System in Cuiabá, state of Mato Grosso. This study showed that exposure to PM_2.5_ had an influence on hospitalizations due to respiratory disease among children under 5 years of age.^18^

Another study on exposure to PM_2.5_ generated by biomass burning and hospitalizations due to respiratory diseases among children and the elderly, which was conducted in two regions of the state of Mato Grosso, presented values of the order of 45 µg/m^3^ in the dry season, with a maximum PM_2.5_ concentration of 260 µg/m^3^. This study showed that an increase in PM_2.5_ concentration of 10 ug/m^3^ resulted in increases in admissions after lags of 3 and 4 days that were of the order of 6% in the dry season. The data accumulated relating to lags of 3 to 5 days showed that the risk increased by 8%.^12^ The CCATT-BRAMS model was also used to estimate the PM_2.5_ concentration in this study. Likewise, a study in Taubaté, SP, which has a population similar to that of Volta Redonda, also found an association between exposure to PM_2.5_, at concentrations similar to those of Volta Redonda, and hospitalizations due to pneumonia and asthma among children.^13^

Chronic and sub-chronic exposure to fine particulate matter, among other pollutants, was shown to be a risk factor for hospitalization due to acute bronchiolitis among children in California.^19^ Using logistic regression, those authors showed that there was an association between exposure to PM_2.5_ and the outcome of hospitalization due to acute bronchiolitis; and that an increase in PM_2.5_ concentration of 10 µg/m^3^ increased the odds of hospitalization by 9% (95% CI: 1.04-1.14). This allowed the authors to suggest that bronchiolitis was one of the adverse effects of exposure to PM_2.5_. Hertz-Picciotto et al.^20^ also identified the adverse effect of exposure to PM_2.5_ and polycyclic aromatic hydrocarbons (PAH) in cohorts of children in two cities in the Czech Republic that had high concentrations of these pollutants. The risk that children under the age of two years would be affected by lower respiratory tract infections due to exposure to PM_2.5_ was higher (RR = 1.30; 95% CI: 1.08-1.58) when the PM_2.5_ concentrations increased by 25 µg/m^3^ and PAH by 100 µg/m^3^.

Sheffield et al.^21^ in the United States found that a 7% decrease in PM_2.5_ levels could result in savings of $ 15 million a year. These authors worked with information from the Nationwide Inpatient Sample relating to the period between 1999 and 2007. There were more than 70 million hospitalizations and 160,000 children under one year of age with a diagnosis of bronchiolitis.

Recently, a study on the role of air pollutants carried out in Volta Redonda found that exposure to these pollutants was responsible for 6% of 5,000 hospitalizations due to diseases in chapter 10 of the ICD-10 between 2005 and 2007, at a cost of $ 170,000 to SUS.^22^

Using the disability adjusted life years (DALY) methodology in relation to reduction of financial cost, exposure to particulate matter was correlated with costs associated with mortality amounting to US$ 1.7 billion annually in 29 metropolitan areas in Brazil.^23^ Ostro and Chestnut^24^ calculated that there would be savings of approximately US$ 70 billion if the average PM_2.5_ concentration were 12 µg/m^3^. It was demonstrated in a study conducted in 211 counties of 51 US metropolitan areas that a decrease in the concentration of this pollutant of 10 ug/m^3^ would increase average life expectancy.^25^

Although the number of hospitalizations in the present study was small, it has to be borne in mind that these related only to hospitalization due to pneumonia, acute bronchitis, bronchiolitis and asthma in all age groups, in a city with around 260,000 inhabitants that was served by SUS hospitals. The damaging effects from exposure in the adult population that led to hospitalization caused by other diseases of the circulatory system, such as hypertension, myocardial infarction and stroke, also has to be taken into consideration.

The mechanisms that lead to pulmonary illnesses are so far poorly understood. Pulmonary and systemic oxidative stress seem to be plausible hypotheses.^26^ Studies have shown that these illnesses involve release of inflammatory mediators, spinal cord stimulation that releases leukocytes and platelets and increased levels of C-reactive protein. Exposure to particulate matter when forest fires occur leads to release of neutrophils and monocytes in addition to production of cytokines by alveolar macrophages. Particulate matter can impair superoxide production by alveolar macrophages, thereby compromising the ability of the lungs to eliminate some of the respiratory tree pathogens.^26^^,^^27^

Riva et al. showed that impairment of pulmonary function occurred in rats after instillation of PM_2.5_. which was translated as lung inflammation, shown by increased activity of myeloperoxidase (MPO), increased influx of neutrophils into the lung parenchyma and increased expression of cytokines like pro-inflammatory TNF-α and IL-6, in addition to oxidative damage.^28^

### Limitations and positive aspects of the study

This study may have limitations; among these, the nature of ecological studies can be highlighted. It was not possible to show the causality between exposure and outcome, but associations between exposures and outcomes could be pointed out. It was not possible to identify whether individuals who were hospitalized had been exposed or whether exposed individuals were admitted. Mistakes in the diagnoses recorded in DATASUS may have led to underreporting or over-notification of cases of pneumonia, asthma, bronchitis and bronchiolitis. Individuals served by private health plans and those treated on an outpatient basis were not included. Another factor that may be considered to be a limitation was that the subjects were not distinguished according to age groups, e.g. as children or elderly people, as done in other studies. Nonetheless, among the total number of hospitalizations shown in our study, 84% were among individuals aged 0 to 10 and over 50 years.

It needs to be pointed out that DATASUS does not provide information about factors associated with the diseases studied here, or about comorbidities. The concentrations were found to be homogeneous throughout the city, and thus exposure to pollutants was assumed to be homogeneous. The pollutant levels were obtained by means of mathematical modeling and good correlation between these data and real data has been identified,^10^ but there may have been some degree of uncertainty concerning the estimation of these data. Nonetheless, it needs to be remembered that use of these data has been recorded in several recent articles^5^^,^^12^^,^^13^^,^^14^ and was very adequate, with few gaps in quantifying the values.

One positive aspect of the present study was that it identified the contribution of exposure to PM_2.5_, which has been little studied in Brazil, towards the number of hospitalizations. The study showed that decreasing the concentration of this pollutant may lead to reduction of the financial cost. DATASUS is an official source of information that has been widely used in studies on the effects of exposure to air pollutants and consequent illnesses and is a trusted source.

## CONCLUSION

This study has shown that a decrease in PM_2.5_ concentration of 5 µg/m^3^ could reduce admissions by up to 76 cases, with a decrease in spending of R$ 84,000 a year. Thus, the results presented here provide support for the city’s healthcare administration towards implementing policies for reducing the levels of air pollution, especially fine particulate material. Such actions would have consequent positive reflections with regard to the number of hospitalizations and expenditure on care provided through these admissions.
